# Lyme Disease as a Potential Precursor to Anti-N-Methyl-D-Aspartate Receptor Encephalitis: A Case Report

**DOI:** 10.7759/cureus.79744

**Published:** 2025-02-27

**Authors:** Cesia G Salan, Aileen De La Rosa, Guillermo Izquierdo-Pretel, Sergey Gerasim

**Affiliations:** 1 Internal Medicine, Ross University School of Medicine, Saint Michael, BRB; 2 Internal Medicine, American University of the Caribbean, Philipsburg, SXM; 3 Hospital Medicine, Jackson Memorial Hospital, Miami, USA; 4 Internal Medicine, Florida International University Herbert Wertheim College of Medicine, Miami, USA; 5 Critical Care Medicine, Jackson Memorial Hospital, Miami, USA

**Keywords:** anti-nmdar encephalitis, anti-tpo, borrelia burgdorferi infection, brief psychosis, clinical diagnosis of lyme disease, white matter changes on mri

## Abstract

Anti-N-methyl-D-aspartate receptor (anti-NMDAR) encephalitis is a complex neuroinflammatory condition often triggered by underlying factors such as viral infections or malignancies, although idiopathic cases are not uncommon. Here, we present a unique case of a 27-year-old female patient diagnosed with anti-NMDAR encephalitis, where Lyme disease emerges as a plausible yet underrecognized trigger.

The patient initially presented to the emergency department (ED) with acute psychosis and was later found to have positive *Borrelia burgdorferi* immunoglobulin G (IgG) titers, antibodies against the NR1 subunit of NMDA, and subacute thyroiditis. A detailed investigation ruled out other paraneoplastic or infectious causes, pointing to Lyme disease as the most likely precipitant. Notably, her exposure to *B. burgdorferi *preceded the onset of encephalitis by two years. Treatment with immunotherapy and antibiotics resulted in significant clinical improvement.

This case highlights the importance of a thorough evaluation of potential triggers of anti-NMDAR encephalitis, emphasizing that it should not be presumed idiopathic without exhaustive investigation. Awareness of Lyme disease as a possible etiological factor may facilitate more targeted management, reduce prolonged hospital stays, and mitigate the burden of extensive diagnostic workups. By addressing the root cause, clinicians can improve patient outcomes and prevent disease recurrence or complications associated with empiric treatment alone.

## Introduction

Anti-N-methyl-D-aspartate receptor (Anti-NMDAR) encephalitis is a severe autoimmune condition characterized by neuropsychiatric symptoms and the presence of immunoglobulin G (IgG) antibodies targeting the NR1 subunit of NMDA receptors. The condition arises from the binding of these autoantibodies to NMDA receptor subunits, leading to receptor internalization, reduced glutamate receptor activity, decreased calcium influx into neurons, and diminished receptor-dependent synaptic currents [1]. Known triggers for the production of these autoantibodies include ovarian teratomas and viral encephalitis, particularly herpes simplex virus (HSV).

In this report, we describe a case where *Borrelia burgdorferi*, the causative agent of Lyme disease, is implicated as a novel trigger for anti-NMDAR encephalitis. Following exposure through a tick bite, the patient developed autoimmune encephalitis. While the specific mechanism underlying excessive intrathecal antibody production remains under investigation, the fundamental pathogenesis-autoantibody production by intrathecal plasma cells parallels that seen in other triggers of anti-NMDAR encephalitis.

Lyme neuroborreliosis, a neurological manifestation of Lyme disease caused by *B. burgdorferi*, typically presents with peripheral neuropathy, facial nerve palsy, radiculitis, and meningitis, with encephalitis being an exceedingly rare complication, present in less than 0.1% of cases of neuroborreliosis. Diagnosis is confirmed via lumbar puncture, revealing elevated intrathecal IgG or IgM antibodies, often accompanied by positive serum antibody titers [2]. Structural abnormalities on magnetic resonance imaging (MRI) may be absent or mimic other conditions, such as multiple sclerosis. Neuroborreliosis may present with a wide spectrum of neuropsychiatric symptoms, which can manifest months to years after initial infection [2].

Most cases of neuroborreliosis are reported in the summer and early fall [3]. In some instances, peripheral nervous system (PNS) manifestations occur before seroconversion, making diagnosis challenging. Both PNS involvement and the rare cases of central nervous system (CNS) inflammation often show rapid improvement with antimicrobial therapy, indicating that active spirochetal infection contributes significantly to the disease process [4]. This case underscores the importance of considering Lyme disease as a potential trigger for autoimmune encephalitis, particularly when other common triggers are absent.

This article was previously presented as a poster at the 2024 Southern Hospital Medicine Conference on September 26, 2024.

## Case presentation

A 27-year-old female patient with no significant past medical history presented to the emergency department (ED) at our tertiary care center in Miami in the spring of 2024 under a Baker Act for an acute psychotic episode. The patient reported two months of intermittent nausea, vomiting, generalized myalgias, fatigue, insomnia, and depression preceding her psychotic episode. Five days before the presentation, she began experiencing non-commanding auditory hallucinations described as "music." At the time of ED admission, the patient was confused, agitated, and unable to recognize family members.

Initial evaluation

A computed tomography (CT) scan of the brain obtained in the ED revealed "symmetric hypodensities along the bilateral anterior internal capsules, nonspecific and unusual for age." The neurological examination was difficult to ascertain due to the patient's agitation requiring chemical sedation. Neurology and Psychiatry were consulted. Neurology recommended brain magnetic resonance imaging (MRI) and electroencephalography (EEG) to rule out seizures as the cause of acute psychosis, while Psychiatry suspected an organic etiology rather than a primary psychiatric disorder.

Hospital course

The timeline of the hospital course of the patient is presented in Table 1.

**Table 1:** Hospital course timeline ESR: erythrocyte sedimentation rate, MRI: magnetic resonance imaging, FLAIR: fluid-attenuated inversion recovery, CT: computed tomography, NMDAR: N-methyl-D-aspartate receptor, EEG: electroencephalography, CSF: cerebrospinal fluid, HSV: herpes simplex virus, TPO: thyroid peroxidase, GCS: Glasgow Coma Scale, AKI: acute kidney injury, IVIG: intravenous immunoglobulin, ICU: intensive care unit, IgG: immunoglobulin G

Hospitalization day	Hospital course
1	The patient was admitted to the medical floor. Initial laboratory results were unremarkable except for a slight elevation in lactic acid and ESR. She remained frequently agitated and combative, requiring chemical restraints and sedation. She was afebrile with no focal neurological deficits and was intermittently drowsy and somnolent due to sedation.
2	The patient became increasingly agitated and was transferred to the intermediate care unit for continuous sedation with dexmedetomidine (Precedex). She developed a fever of unknown origin and was empirically started on vancomycin and cefepime. Brain MRI revealed T2 FLAIR signal hyperintensities involving the bilateral ganglio-capsular structures, surrounding white matter, left thalamus, and genu of the corpus callosum (Figure 1).
5	The patient remained febrile. Abdominal and pelvic CT was performed to rule out malignancy as a potential source of fever and revealed a cystic lesion in the left ovary. Anti-NMDAR encephalitis secondary to ovarian teratoma was considered, but a follow-up transvaginal ultrasound revealed a simple cyst with no signs of malignancy. EEG showed no epileptiform activity. Lumbar puncture revealed clear CSF with normal opening pressure, increased lymphocytes, and increased total cell count, making an infectious cause less likely. CSF viral and bacterial tests, including HSV and syphilis, were negative at this time as well. Anti-TPO antibodies were elevated at 270 IU/mL, consistent with subclinical thyroiditis. Thyroid ultrasound revealed a thyroid mildly heterogeneous in echogenicity without focal mass or calcifications. Hashimoto's encephalopathy was added to the differential at this time due to these results.
6	The patient's mental status declined further; she no longer followed commands and spoke in nonsensical terms. The GCS score was 11/15 (eyes: 4 for spontaneous movement of eyes, verbal: 2 for incomprehensible sounds, motor: 5 for movement localized to pain). High-dose intravenous methylprednisolone (Solu-Medrol 1 g daily for five days) was initiated for presumed encephalitis of unknown origin.
12	After no improvement with steroids, plasmapheresis was initiated (six sessions planned over 10-14 days). Hashimoto's encephalopathy was ruled out due to the patient's lack of response to steroid treatment. The hospital course was complicated by vancomycin-induced AKI requiring renal replacement therapy and hemodialysis.
13	Telemetry revealed intermittent narrow complex tachycardia with heart rates up to 254 beats per minute. Cardiology recommended electrophysiological studies. Infectious disease documented that encephalitis of unknown origin remained the primary concern, with all blood and CSF cultures as well as routine autoimmune workups returning negative results. CSF autoimmune encephalitis panel was pending.
22	Following the completion of plasmapheresis, minimal improvement in mentation was observed. A repeat brain MRI revealed an evolving left internal capsular lacunar infarction with prior insult/ischemia to the right internal capsule but no acute infarctions.
26	The patient exhibited dysautonomia and tachy-bradyarrhythmia syndrome, with heart rates ranging from 30 to 150 beats per minute and sinus pauses up to 7.2 seconds. Cardiology recommended Lyme titers to evaluate for Lyme carditis.
29	Neurology recommended IVIG therapy (2 g/kg over five days) for presumed autoimmune encephalitis of unknown etiology. CSF autoimmune panel results were still pending.
38	Despite an exhaustive infectious, neoplastic, and paraneoplastic workup, no conclusive diagnosis was reached. A detailed family interview revealed that the patient had traveled to the northeastern United States two years prior, prompting testing for *Borrelia burgdorferi* antibodies.
40	CSF autoimmune encephalitis panel returned positive for antibodies against the NR1 subunit of NMDAR, confirming a diagnosis of anti-NMDAR encephalitis. The patient was transferred to the neuro-ICU to determine eligibility for the Extinguish Trial. Following confirmation of the anti-NMDAR encephalitis diagnosis, the patient was enrolled in a double-blind clinical trial for the treatment of anti-NMDAR encephalitis, with treatment administered in accordance with the trial protocol. Due to the double-blind nature of the trial, the specific treatment received remains unknown.
42	Repeat brain MRI demonstrated significant interval resolution of previously noted restricted diffusion in the left anterior internal capsule with minimal residual FLAIR hyperintensity. The patient was enrolled in the Extinguish Trial and received her first infusion. There was no change in mentation at this point, and the patient remained somnolent and intermittently agitated.
43	A repeat transvaginal ultrasound showed a fibroid uterus and normal ovaries, with no cysts identified. The previously noted ovarian cyst was deemed a follicular cyst. Other potential etiologies for anti-NMDAR encephalitis were ruled out.
53	Lyme titers returned positive for IgG antibodies against *B. burgdorferi*, confirming latent Lyme disease. Infectious disease recommended a seven-day course of intravenous ceftriaxone (2 g daily). IVIG and Extinguish Trial infusions continued.
60	Following antibiotic therapy and continued treatment for anti-NMDAR encephalitis (IVIG and Extinguish Trial), the patient demonstrated robust clinical improvement. She became oriented to person, place, and time, and was able to follow commands. Her family reported that she had returned to baseline mentation.
67	The patient completed her IVIG course and was discharged home in stable condition.

**Figure 1:**
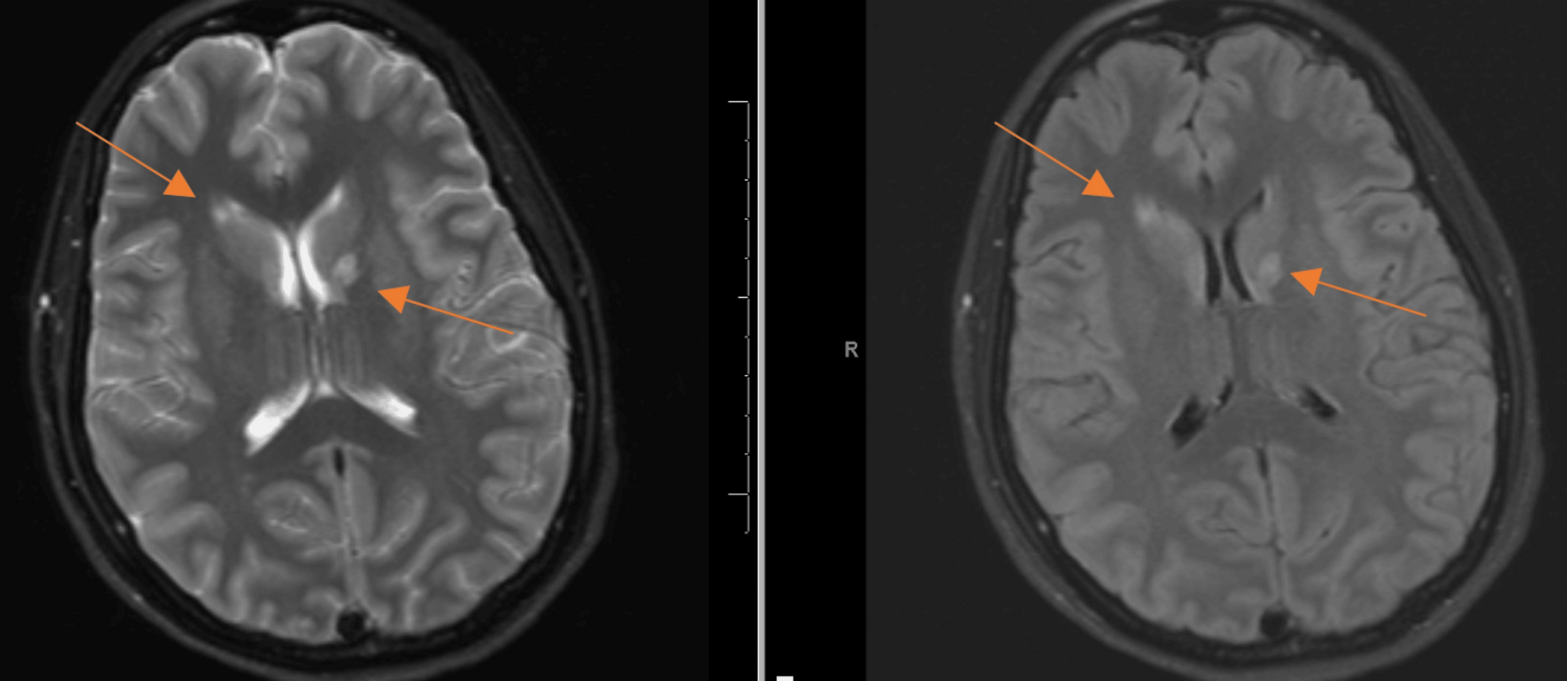
Brain MRI with and without contrast at the initial presentation demonstrating T2 FLAIR hyperintensities in the bilateral ganglio-capsular structures and surrounding white matter, with additional involvement of the left thalamus and the genu of the corpus callosum (arrows) MRI: magnetic resonance imaging, FLAIR: fluid-attenuated inversion recovery

Figure 1 shows the brain MRI of the patient at the initial presentation. The laboratory result of the patient is shown in Table 2.

**Table 2:** Pertinent laboratory values TPO: thyroid peroxidase, CSF: cerebrospinal fluid, IgG: immunoglobulin G, IgM: immunoglobulin M

Laboratory test	Laboratory value	Reference range
Lactic acid	2.5 mmol/L	0.7-2.1 mmol/L
Erythrocyte sedimentation rate	33 mm/hour	0-30 mm/hour
Anti-TPO antibody	270 IU/mL	<30 IU/mL
CSF lymphocytes %	91%	40%-80%
CSF total cell count	14 mm^3^	0-5 mm^3^
CSF anti-NR1	Positive	Negative
IgG *B. burgdorferi* (Western blot confirmatory testing)	Positive	Negative
IgM *B. burgdorferi* (Western blot confirmatory testing)	Negative	Negative

Definitive management/outcome and follow-up

The combined treatment of intravenous immunoglobulin (IVIG) and ceftriaxone resulted in significant clinical and radiological improvement in our patient, with expectations for a full recovery. Counseling and education were provided to the patient's family regarding the importance of rehabilitation, including physical and occupational therapy. A tailored in-home exercise program was recommended to support the patient's continued recovery, focusing on regaining strength, coordination, and functional independence. Regular follow-up appointments were scheduled to monitor for any signs of recurrence and to assess the patient's neurological and psychological progress.

## Discussion

Anti-N-methyl-D-aspartate receptor (anti-NMDAR) encephalitis is an autoimmune-mediated inflammation of brain tissue caused by antibodies targeting the NR1 subunit of the NMDA receptor. This disease can arise from both paraneoplastic and non-paraneoplastic triggers. Paraneoplastic encephalitis is associated with antibodies directed against intracellular and extracellular proteins, often linked to teratomas, whereas non-paraneoplastic cases are typically idiopathic or postinfectious, with herpes simplex virus encephalitis being a well-documented trigger. Patients often present with prodromal symptoms and psychiatric manifestations, making timely diagnosis essential. Diagnosis is best achieved through cerebrospinal fluid (CSF) analysis, which is more sensitive and specific than serum testing [5,6]. Delays in initiating immunosuppressive therapy can worsen outcomes and are associated with hippocampal damage [7]. The presented case adds to the growing body of evidence by identifying a novel etiology after a comprehensive infectious and paraneoplastic workup excluded established triggers of autoimmune encephalitis.

Lyme disease, caused by tick-borne* Borrelia* species, is endemic to certain regions, with *Borrelia burgdorferi* being predominant in the northeastern United States [3]. Lyme disease manifests with a broad clinical spectrum, ranging from flu-like symptoms in the prodromal stage to severe neuropsychiatric presentations in later stages, including those resembling schizophrenia and bipolar disorder [8]. Studies suggest that interactions between damaged neurons and dysregulated, overactive microglia can create a self-sustaining inflammatory process, driving the pathophysiology of the disease [4]. In cases involving both peripheral and central nervous system inflammation, rapid improvement following antimicrobial therapy provides compelling evidence for the direct involvement of the spirochete in the disease process [9].

The association between Lyme disease and anti-NMDAR encephalitis is exceedingly rare, with only two prior case reports: one involving a 74-year-old man and another a 32-year-old man [10]. Our case underscores the need to consider Lyme disease as a potential trigger for anti-NMDAR encephalitis, especially when conventional causes are excluded. Efficient resource allocation is crucial in such cases to expedite diagnosis and optimize care.

Importantly, Lyme disease can elicit multiple autoimmune responses beyond encephalitis. Our patient also exhibited thyroiditis, which aligns with evidence that *Borrelia* spirochetal proteins share antigenic properties with thyroid-related proteins such as the TSH receptor, thyroglobulin, and thyroid peroxidase. This molecular mimicry can trigger thyroid autoimmunity, resulting in conditions such as Hashimoto's thyroiditis, silent thyroiditis, or Hashitoxicosis [11]. Our patient's subclinical thyroiditis, evidenced by elevated anti-TPO levels and heterogeneous thyroid ultrasound findings, aligns with existing literature and highlights the broader autoimmune potential of untreated Lyme disease.

By identifying the underlying etiology of autoimmune encephalitis, clinicians can improve both patient outcomes and hospital triage reliability. Further investigation into the relationship between Lyme disease and autoimmune diseases such as anti-NMDAR encephalitis is essential to enhance diagnostic accuracy, streamline treatment approaches, and reduce the burden of extensive diagnostic evaluations.

## Conclusions

This case underscores the importance of considering Lyme disease as a potential trigger in neuropsychiatric presentations and in cases of anti-NMDAR encephalitis, even when geographic location or clinical history does not strongly support exposure. After ruling out common triggers of anti-NMDAR encephalitis, a systematic review of the literature revealed only two prior reports linking Lyme disease as the sole precipitant of this condition. This highlights the critical need to treat with both antimicrobials and immunotherapy in such cases.

Failure to address underlying Lyme disease can lead to worsening of the clinical presentation. In our patient, significant improvement was observed only after initiating antimicrobial therapy for Lyme disease, as prior treatments for encephalitis alone had failed to improve her mentation. This case further emphasizes the need for early consideration of Lyme disease in the differential diagnosis to avoid prolonged and costly hospitalizations. Finally, late diagnosis and treatment of Lyme disease can result in additional autoimmune complications, including myocarditis and thyroiditis. Clinicians should maintain a high index of suspicion and adopt a comprehensive diagnostic approach to optimize outcomes and prevent complications associated with delayed treatment.
